# Mortality associated with metabolic syndrome in people with COPD managed in primary care

**DOI:** 10.1183/23120541.00211-2022

**Published:** 2022-10-24

**Authors:** Urvee Karsanji, Rachael A. Evans, Jennifer K. Quint, Kamlesh Khunti, Claire A. Lawson, Emily Petherick, Neil J. Greening, Sally J. Singh, Matthew Richardson, Michael C. Steiner

**Affiliations:** 1 NIHR Leicester Biomedical Research Centre – Respiratory, Dept of Respiratory Sciences, College of Life Sciences, University of Leicester, Leicester, UK; 2 National Heart and Lung Institute, Imperial College London, London, UK; 3 NIHR Applied Research Collaboration East Midlands, Diabetes Research Centre, University of Leicester, Leicester, UK; 4 Dept of Cardiovascular Sciences, University of Leicester, Leicester, UK; 5 School of Sport, Exercise and Health Sciences, Loughborough University, Loughborough, UK

## Abstract

**Objective:**

The prevalence of metabolic syndrome (MetS) has been reported to be higher in selected populations of people with COPD. The impact of MetS on mortality in COPD is unknown. We used routinely collected healthcare data to estimate the prevalence of MetS in people with COPD managed in primary care and determine its impact on 5-year mortality.

**Methods:**

Records from 103 955 patients with COPD from the Clinical Practice Research Datalink (CPRD-GOLD) between 2009 to 2017 were scrutinised. MetS was defined as the presence of three or more of: obesity, hypertension, lowered high-density lipoprotein cholesterol, elevated triglycerides or type 2 diabetes mellitus (T2DM). Univariate and multivariable Cox regression models were constructed to determine the prognostic impact of MetS on 5-year mortality. Similar univariate models were constructed for individual components of the definition of MetS.

**Results:**

The prevalence of MetS in the COPD cohort was 10.1%. Univariate analyses showed the presence of MetS increased mortality (hazard ratio (HR) 1.19, 95% CI: 1.12–1.27, p<0.001), but this risk was substantially attenuated in the multivariable analysis (HR 1.06, 95% CI: 0.99–1.13, p*=*0.085). The presence of hypertension (HR 1.70, 95% CI: 1.63–1.77, p<0.001) and T2DM (HR 1.41, 95% CI: 1.34–1.48, p<0.001) increased and obesity (HR 0.74, 95% CI: 0.71–0.78, p<0.001) reduced mortality risk.

**Conclusion:**

MetS in patients with COPD is associated with higher 5-year mortality, but this impact was minimal when adjusted for indices of COPD disease severity and other comorbidities. Individual components of the MetS definition exerted differential impacts on mortality suggesting limitation to the use of MetS as a multicomponent condition in predicting outcome in COPD.

## Abstract 2

**The presence of MetS in COPD is not significantly associated with increased mortality over 5 years. The individual components of MetS exert differential effects on mortality, limiting the utility of the syndrome in predicting outcome.** https://bit.ly/3PooGQd

## Introduction

COPD is recognised to have a number of systemic consequences beyond the primary pulmonary pathophysiology and is frequently associated with a high prevalence of multiple comorbid long-term conditions. Metabolic syndrome (MetS) describes the coexistence of linked metabolic pathophysiologies known to be associated with adverse health outcomes, principally resulting from the development of type 2 diabetes mellitus (T2DM) and cardiovascular disease (CVD) [1]. A systematic review estimated the prevalence of MetS in COPD at 32% (95% CI: 25–58), which was higher than control populations without COPD (prevalence 30%; 95% CI: 17–54) [2]. However, the majority of published prevalence studies in COPD scrutinised selected cohorts from hospital clinics, rehabilitation programmes or acute admissions, and the true population prevalence across the range of COPD severities (the majority of which will be managed in primary care) is uncertain.

Accepted definitions of MetS comprise several component indices, typically abdominal obesity, dyslipidaemia, hypertension and hyperglycaemia [3]. Whilst these indices are relatively easy to measure, they are frequently not routinely recorded in primary care [4], thus presenting a challenge for the estimation of MetS prevalence in primary care clinical populations.

The presence of MetS in people with COPD is of potential prognostic and functional importance because COPD itself confers an increased risk of cardiovascular mortality, and indices incorporated in the syndrome (such as obesity) may have a significant impact on symptom burden, activity limitation and longer-term outcomes [5]. However, the impact of MetS on mortality in COPD has not previously been reported. Importantly, its prognostic impact may not mirror that of the non-COPD population because of the well-documented protective effect of increased body mass index (BMI) in COPD, referred to as the “obesity paradox” [6].

In this study, we sought firstly to estimate the prevalence of MetS in a general population recorded as having COPD in primary care, using a modified definition of MetS developed and tested for the purpose of estimating prevalence from routinely recorded primary care clinical records (Aim 1) [7]. Secondly, we investigated the association of MetS in people with COPD with disease severity, symptom burden (breathlessness and exacerbations) and comorbidities (Aim 2). Thirdly, we determined the association of the presence of MetS (and its component indices) on subsequent all-cause 5-year mortality in COPD (Aim 3).

## Methods

### Study design and population

We conducted a retrospective longitudinal cohort study using routinely collected healthcare data provided by the Clinical Practice Research Datalink (CPRD-GOLD, July 2019 build), the largest source of electronic primary care data representing 5% of people in the general UK population [8, 9]. Linked pseudonymised mortality data from the Office for National Statistics (ONS) was provided for this study by CPRD for patients in England. Data are linked by NHS Digital, the statutory trusted third party for linking data, using identifiable data held only by NHS Digital. Selected general practices consent to this process at a practice level, with individual patients having the right to opt out. Hospital admission data were linked through Hospital Episode Statistics (HES), and deprivation quintiles from the English Index of Multiple Deprivation (IMD). Ethics approval was obtained from ISAC (the Independent Scientific Advisory Committee overseeing CPRD (Protocol No. 18_138R)).

The study population were patients over the age of 35 years with COPD, and alive and registered between the study-interval dates from 1 January 2009 to 31 July 2017 with a minimum of 12 months data prior to study entry. All included patients had a coded COPD diagnosis determined using pre-specified medcodes and validated definitions (see supplementary appendix S1) [10].

Using a previously reported definition with information available from primary care records [7], MetS was defined as the presence of at least three of the following five conditions:

- Obesity (defined as BMI at least 30 kg·m^−2^)
- Hypertension
- Lowered high-density lipoprotein (HDL) cholesterol (<40 mg·dL^−1^ (men); <50 mg·dL^−1^ (women))
- Elevated triglycerides (TG) (>150 mg·dL^−1^)
- Presence of type 2 diabetes mellitus (T2DM)

Other comorbidities were categorised into groups based on their relevance to clinical outcome and health status from previous studies [11]. Categories included liver disease, kidney disease, psychological disorder (interpreted as one or more of anxiety, depression or dementia), frailty (one or more of malnutrition, poor vision, incontinence, a history of falls, Social Services involvement), asthma, cardiovascular disease (one or more of heart failure, stroke, myocardial infarction or atrial fibrillation), a musculoskeletal condition (one or more of rheumatoid arthritis, osteoarthritis, osteoporosis) and gastroesophageal reflux disorder (GERD). These comorbidities were identified using medcodes in CPRD and found using International Classification of Disease 10th Revision (ICD-10) codes in HES data at any point prior to COPD index date.

### Outcomes

All-cause 5-year mortality was derived from ONS data. ONS derives the underlying cause of death from death certificates and is coded using ICD-10 codes. Mortality rates were compared between patients with and without MetS, and survival curves over 5-year follow-up were plotted.

### Covariates

Supplementary table S1k lists and describes candidate variables which were assigned to demographic, COPD-specific (list) or comorbidity groups together with predicted outcomes of all-cause mortality. COPD-specific covariates were continuous, whereas comorbidities were binary and related to the presence or absence of conditions.

Acute exacerbation of COPD (AECOPD) was expected to be a key predictor for mortality, and we therefore recorded these events in the 12 months prior to case ascertainment, defined using a combination of diagnostic and therapy codes that have been shown to be valid [12] (see supplementary table S1c). Based on GP records, we therefore defined AECOPD as any of the following:

- antibiotic (ABX) and steroid (OCS) prescriptions prescribed for 5–14 days,
- two out of three symptoms from cough, sputum, breathlessness and ABX or OCS for 5–14 days,
- a lower respiratory tract infection,

or from hospital data COPD as primary diagnosis.

### Statistical analyses

The prevalence of MetS was estimated using descriptive statistics. For baseline characteristics, we assessed demographics of the population using measures of central tendency and variances appropriate to their respective distributions, *i.e.* mean±sd. For categorical variables, the number of patients (n) belonging to each category and percentage (%) were given. Patient characteristics were compared between those defined as having MetS and those not having MetS.

Multiple logistic regression (LR) models were developed to determine the association between clinical and demographic factors and the presence of MetS. Variables included were: sex, age, ethnicity, smoking status, IMD quintile, forced expiratory volume in 1 s (FEV_1_ % predicted), exacerbation frequency, Medical Research Council (MRC) score and comorbidity classification (see supplementary table S1j). Receiver operating characteristic (ROC) area under the curve (AUC) statistics were used to determine the best cut-off value for predicting whether a new patient was likely to present with or without MetS.

Kaplan–Meier survival curves were plotted for COPD patients with and without MetS, and also with and without the individual component conditions of MetS (obesity, hypertension, lowered HDL cholesterol, elevated TG, presence of T2DM). Univariate and multivariable Cox proportional hazard ratios were calculated for 5-year survival for patients with and without MetS and the individual component conditions of MetS. Covariates chosen for the multivariable analysis were those deemed statistically significant (p<0.001) in the univariate multiple LR models outlined for Aim 2.

Multivariable associations were also explored as part of a sensitivity analysis between MetS and all-cause 5-year mortality when stratified by:

1. Demographics: sex, age, ethnicity
2. IMD quintile: (1 being least deprived and 5 being most deprived)
3. COPD severity: MRC dyspnoea grade, FEV_1_ % predicted, smoking status, number of exacerbations
4. Comorbidities: liver disease, kidney disease, psychological disease, frailty, asthma, CVD, musculoskeletal disease, GERD

## Results

**FIGURE 1:**
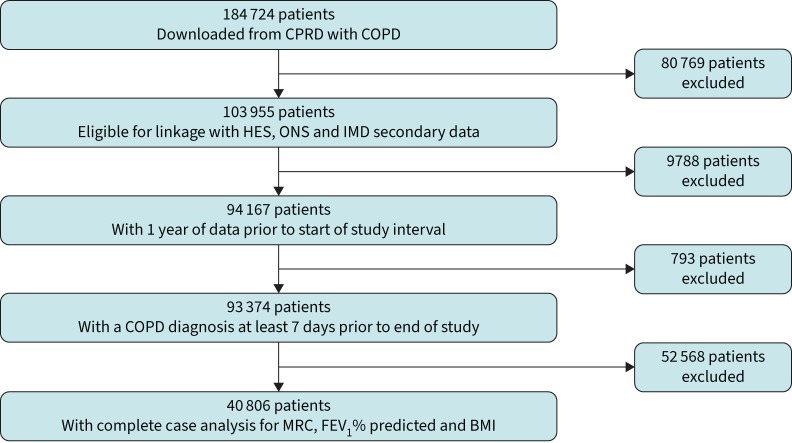
Flowchart identifying the cohort of COPD patients used to apply the components of the metabolic syndrome (MetS) definition using complete case analysis for the components of MetS and disease predictors: Medical Research Council (MRC) score, forced expiratory volume in 1 s (FEV_1_) % predicted and body mass index (BMI). The variables FEV_1_ % predicted, BMI and MRC grade each had over 25% missingness, hence were excluded from the cohort and full-case analysis was used leaving a generous sample of n=40 806 patients. CPRD: Clinical Practice Research Datalink; HES: Hospital Episode Statistics; ONS: Office for National Statistics; IMD: Index of Multiple Deprivation.

We included 40 806 patients with COPD who met eligibility criteria (figure 1), of whom 54.3% were male and 45.7% were female, with an overall mean±sd age of 69±11 years and mean±sd BMI 27.6±6 kg·m^−2^. Summary statistics are shown in table 1. Mean FEV_1_ % predicted was 63.1 but with wide variation (sd=23), confirming a broad range of disease severity.

**TABLE 1:** Baseline characteristics for the population defined by the presence of metabolic syndrome (MetS)

	**All**	**MetS**	**Non-MetS**
**Subjects n**	40 806	4141	36 665
**Sex**			
Male	22** **165 (54.32)	2421 (58.46)	19** **744 (53.85)
Female	18** **641 (45.68)	1720 (41.54)	16** **921 (46.15)
**Ethnicity**			
Black	196 (0.48)	23 (0.56)	173 (0.47)
Mixed other	216 (0.53)	22 (0.53)	194 (0.53)
South Asian	416 (1.02)	70 (1.69)	346 (0.94)
Unknown	1742 (4.27)	55 (1.33)	1687 (4.60)
White	38** **236 (93.70)	3971 (95.89)	34** **265 (93.45
**Age years, mean±sd**	68.69±10.74	68.92±9.60	68.66±10.87
35–44	675 (1.65)	49 (1.18)	626 (1.71)
45–54	3659 (8.97)	281 (6.79)	3378 (9.21)
55–64	9431 (23.11)	973 (23.50)	8458 (23.07)
65–74	14** **228 (34.87)	1597 (38.57)	12** **631 (34.45)
75+	12** **813 (31.40)	1241 (29.97)	11** **572 (31.56)
**BMI kg·m^−2^, mean±sd**	27.56±6.02	34.10±5.66	26.82±5.60
Underweight (<18.5)	1546 (3.79)	6 (0.14)	1540 (4.20)
Normal (18.5–24.9)	13** **168 (32.27)	169 (4.08)	12** **999 (35.45)
Overweight (25–29.9)	13** **719 (33.62)	474 (11.45)	13** **245 (36.12)
Obese (30–39.9)	10** **929 (26.78)	2926 (70.66)	8003 (21.83)
Severely obese (≥40)	1444 (3.54)	566 (13.67)	878 (2.39)
**Smoking status**			
Current smoker	14** **532 (35.61)	1147 (27.70)	13** **385 (36.51)
Nonsmoker/never	4544 (11.14)	501 (12.10)	4043 (11.03)
Ex-smoker	21** **011 (51.49)	2448 (59.12)	18** **563 (50.63)
Missing	719 (1.76)	45 (1.09)	674 (1.84)
**Index of multiple deprivation**			
1=least deprived	6331 (15.51)	559 (13.50)	5772 (15.74)
2	7581 (18.58)	684 (16.52)	6897 (18.81)
3	7797 (19.11)	771 (18.62)	7026 (19.16)
4	9152 (22.43)	950 (22.94)	8202 (22.37)
5=most deprived	9945 (24.37)	1177 (28.42)	8768 (23.91)
**FEV_1_ % predicted, mean±sd**	63.11±22.76	64.25±20.56	62.98±23.00
**Number of exacerbations in previous year**			
0	26 773 (65.61)	2678 (64.67)	24 095 (65.72)
1	9191 (22.52)	912 (22.02)	8279 (22.58)
2	3005 (7.36)	342 (8.26)	2663 (7.26)
3	1086 (2.66)	118 (2.85)	968 (2.64)
4	395 (0.97)	51 (1.23)	344 (0.94)
5	212 (0.52)	26 (0.63)	186 (0.51)
6	89 (0.22)	11 (0.27)	78 (0.21)
7+	55 (0.13)	3 (0.07)	52 (0.14)
**MRC dyspnoea score**			
1=least severe	7928 (19.43)	568 (13.72)	7360 (20.07)
2	16** **688 (40.90)	1458 (35.21)	15** **230 (41.54)
3	10** **161 (24.90)	1191 (28.76)	8970 (24.46)
4	5173 (12.68)	793 (19.15)	4380 (11.95)
5=most severe	856 (2.10)	131 (3.16)	725 (1.98)
**GOLD, n (%)**			
I	8577 (21.02)	837 (20.21)	7740 (21.11)
II	19** **776 (48.46)	2248 (54.29)	17** **528 (47.81)
III	10** **150 (24.87)	935 (22.58)	9215 (25.13)
IV	2303 (5.64)	121 (2.92)	2182 (5.95)
**Components of MetS definition**			
Obesity (BMI at least 30)	12** **373 (30.32)	3492 (84.33)	8881 (24.22)
Hypertension	14** **846 (36.38)	3515 (84.88)	11** **331 (30.90)
HDL (<40 mg·dL^−1^ (men); <50 mg·dL^−1^ (women))	6231 (15.27)	2862 (69.11)	3369 (9.19)
TG (>150 mg·dL^−1^)	595 (1.46)	418 (10.09)	177 (0.48)
T2DM	6967 (17.07)	3255 (78.60)	3712 (10.12)
**Comorbidities**			
Liver disease	982 (2.41)	196 (4.73)	786 (2.14)
Kidney disease	5845 (14.32)	1049 (25.33)	4796 (13.08)
Psychological disease	4258 (10.43)	554 (13.38)	3704 (10.10)
Frailty	1514 (3.71)	229 (5.53)	1285 (3.50)
Asthma	16** **315 (39.98)	1886 (45.54)	14** **429 (39.35)
CVD	7416 (18.17)	1415 (34.17)	6001 (16.37)
Musculoskeletal	8972 (21.99)	1316 (31.78)	7656 (20.88)
GERD	3581 (8.78)	471 (11.37)	3110 (8.48)

Categorical data presented as n (%) unless otherwise indicated. BMI: body mass index; FEV_1_: forced expiratory volume in 1 s; MRC: Medical Research Council; GOLD: Global Initiative for Chronic Obstructive Lung Disease; HDL: high-density lipoprotein; TG: triglyceride; T2DM: type 2 diabetes mellitus; CVD: cardiovascular disease; GERD: gastroesophageal reflux disorder.

The prevalence of MetS in the COPD cohort was 10.1%. Age and sex were comparable for patients presenting with and without MetS. Over 90% of patients belonged to a White background irrespective of MetS status. Neighbourhood deprivation was analogous for quintiles 1–4, however, there appear to be more adults in the highest deprivation quintile (IMD=5) with MetS compared to without (28.4% *versus* 23.9%). Full demographics and disease characteristics for those with and without MetS are shown in table 1. The most prevalent qualifying components of MetS were hypertension (84.9%) and obesity (84.3%), and least prevalent was elevated TG (10.1%). The three most frequent combinations of components needed to meet the definition of MetS revealed over 50% (N=2073) of patients with obesity, hypertension and T2DM.

A multiple LR model identified clinical and demographic predictors most associated with MetS to be sex (male), age, current smokers, missing smoking status, IMD5 (most severe), FEV_1_ % predicted, MRC grades 2–5, liver disease, kidney disease, frailty, asthma, CVD and musculoskeletal disease. These were all deemed statistically significant using a threshold of p<0.001, as shown in table 2. Predictors identified as statistically significant were then used in another multivariable LR model to determine if there is still an association between them and the identification of MetS in patients with COPD. An AUC value of 0.688 was generated showing the trade-off between sensitivity and specificity, indicating moderate prediction performance for identification of MetS patients (ROC AUC statistics can be seen in supplementary figure S2a).

**TABLE 2:** Multiple associations using the odds ratio (OR) between disease and demographic variables and the presence of metabolic syndrome (MetS)

**Variable**	**OR**	**p-value**
**genderFemale**	ref.	
**genderMale**	1.20	**<0.001**
**age**	0.98	**<0.001**
**ethnicityWhite**	ref.	
**ethnicityBlack**	1.15	0.551
**ethnicityMixed_Other**	0.99	0.970
**ethnicitySouth Asian**	1.70	**<0.001**
**ethnicityUnknown**	0.43	**<0.001**
**smokingNon**	ref.	
**smokingEx**	1.05	0.337
**smokingCurrent**	0.68	**<0.001**
**smokingMissing**	0.58	**<0.001**
**imd1**	ref.	
**imd2**	1.00	0.947
**imd3**	1.09	0.156
**imd4**	1.15	0.137
**imd5**	1.32	**<0.001**
**fev1pp**	1.00	**<0.001**
**n_exacs**	0.96	0.027
**mrc1**	ref.	
**mrc2**	1.21	**<0.001**
**mrc3**	1.57	**<0.001**
**mrc4**	2.02	**<0.001**
**mrc5**	1.97	**<0.001**
**liver**	1.74	**<0.001**
**kidney**	1.79	**<0.001**
**psych**	1.15	0.006
**frail**	1.32	**<0.001**
**asthma**	1.16	**<0.001**
**cvd**	2.22	**<0.001**
**mskel**	1.51	**<0.001**
**gerd**	1.08	0.139

Variables highlighted in bold are associated with the presence of MetS.

**FIGURE 2:**
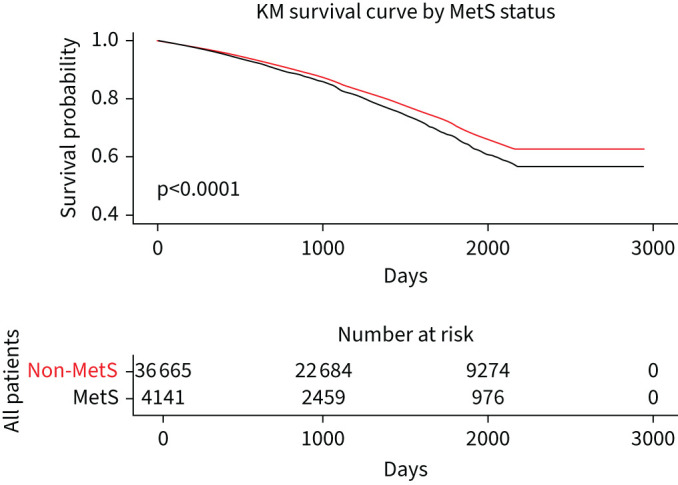
The association of the presence of metabolic syndrome (MetS) with all-cause mortality. KM: Kaplan–Meier.

**FIGURE 3:**
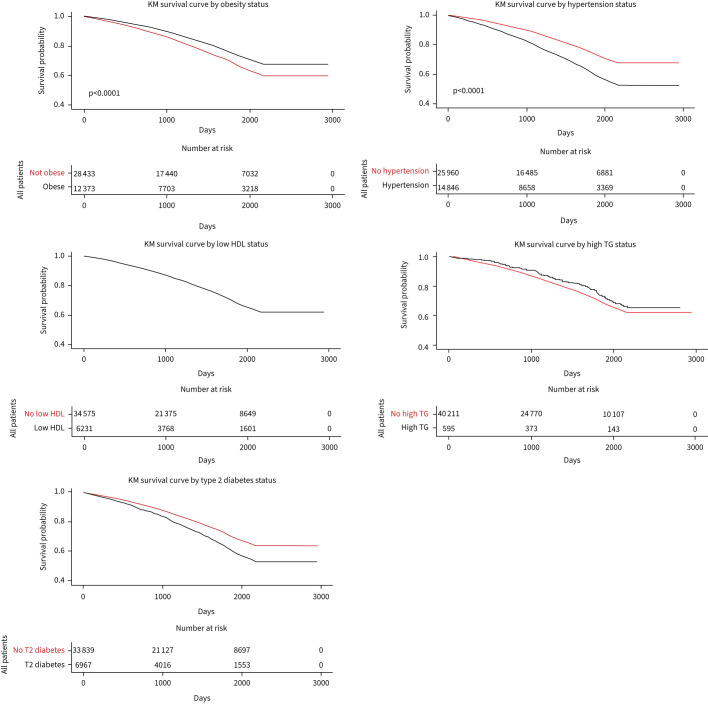
Kaplan–Meier (KM) survival curves showing survival probability by component of metabolic syndrome (MetS) definition: obesity, high-density lipoprotein (HDL) status, triglyceride status (TG), hypertension and type 2 (T2) diabetes mellitus.

The association of the presence of MetS with all-cause mortality is shown in figure 2, and all-cause mortality for the individual components of MetS is plotted in figure 3a–e. Univariate all-cause 5-year mortality hazard ratios (HRs) for the presence of MetS and components of the MetS definition are show in table 3. The presence of MetS was associated with a significantly higher risk of mortality (HR 1.19, 95% CI: 1.12–1.27), and the main traits from the definition associating with higher mortality were hypertension (HR 1.70, 95% CI: 1.63–1.77) and T2DM (HR 1.41, 95% CI: 1.34–1.48). Obesity was associated with a lower mortality (HR 0.74, 95% CI: 0.71–0.78).

**TABLE 3:** Hazard ratios (HRs) for all-cause mortality for the presence of metabolic syndrome and individual components

**Variable**	**HR (95% CI)**	**p-value**
**mets**	1.19 (1.12–1.27)	**<0.001**
**obese**	0.74 (0.71–0.78)	**<0.001**
**hyp**	1.70 (1.63–1.77)	**<0.001**
**lowhdl**	1.00 (0.95–1.06)	0.885
**hightg**	0.83 (0.69–0.99)	0.041
**diabetesT2**	1.41 (1.34–1.48)	**<0.001**

Variables highlighted in bold are associated with 5-year all-cause mortality.

The univariate associations between individual components of the MetS variables (see Aim 2) and 5-year survival can be found in supplementary table S2a. Table 4 shows the multivariable associations of these same variables with 5-year survival. In contrast to univariate analysis, MetS was not significantly associated with mortality in the multivariable analysis when considering multiple predictors (HR 1.06, 95% CI: 0.99–1.13).

**TABLE 4:** Multivariable hazard ratio (HR) model between adjustment variables and 5-year all-cause mortality

**Variable**	**HR (95% CI)**	**p-value**
**mets=0**	ref	-
**mets=1**	1.06 (0.99–1.13)	0.085
**genderFemale**	ref	-
**genderMale**	1.33 (1.27–1.38)	**<0.001**
**age**	1.07 (1.07–1.07)	**<0.001**
**ethnicityWhite**	ref	-
**ethnicityBlack**	0.63 (0.42–0.93)	0.020
**ethnicityMixed_Other**	0.70 (0.55–0.90)	0.005
**ethnicitySouth Asian**	0.99 (0.70–1.41)	0.970
**ethnicityUnknown**	0.84 (0.72–0.97)	0.020
**smokingNon**	ref	-
**smokingEx**	1.17 (1.10–1.26)	**<0.001**
**smokingCurrent**	1.75 (1.62–1.89)	**<0.001**
**smokingMissing**	1.16 (1.00–1.35)	0.044
**imd1**	ref	-
**imd2**	1.05 (0.98–1.12)	0.190
**imd3**	1.06 (0.99–1.14)	0.102
**imd4**	1.05 (0.98–1.12)	0.193
**imd5**	1.13 (1.05–1.20)	**<0.001**
**fev1pp**	0.99 (0.99–0.99)	**<0.001**
**n_exacs**	1.08 (1.06–1.10)	**<0.001**
**mrc1**	ref	-
**mrc2**	1.21 (1.12–1.29)	**<0.001**
**mrc3**	1.53 (1.42–1.64)	**<0.001**
**mrc4**	1.89 (1.75–2.04)	**<0.001**
**mrc5**	2.88 (2.57–3.22)	**<0.001**
**liver**	1.53 (1.36–1.72)	**<0.001**
**kidney**	1.25 (1.19–1.32)	**<0.001**
**psych**	1.41 (1.32–1.51)	**<0.001**
**frail**	1.26 (1.15–1.38)	**<0.001**
**asthma**	0.93 (0.89–0.97)	**<0.001**
**cvd**	1.44 (1.37–1.51)	**<0.001**
**mskel**	1.02 (0.98–1.07)	0.350
**gerd**	0.99 (0.91–1.06)	0.700

Variables highlighted in bold are associated with 5-year all-cause mortality.

Table 5 highlights the effect of MetS on all-cause mortality after stratification by demographics, deprivation, COPD severity and comorbidities. In comparison to the crude HR for MetS on mortality (HR 1.19), comorbidities reduced the effect of MetS on mortality (HR 0.95, 95% CI: 0.89–1.01), whereas the other three stratification classifications all were comparable to the individual effect.

**TABLE 5:** Stratified multivariable survival model on 5-year all-cause mortality by the presence of metabolic syndrome

**Adjusted for**	**Variable**	**HR (95% CI)**	**p-value**
**Demographics**	mets=1	1.17 (1.10–125)	**<0.001**
**IMD**	mets=1	1.19 (1.12–1.27)	**<0.001**
**COPD severity**	mets=1	1.13 (1.02–1.25)	0.021
**Comorbidities**	mets=1	0.95 (0.89–1.01)	0.100

HR: hazard ratio; IMD: Index of Multiple Deprivation. Variables highlighted in bold are associated with 5-year all-cause mortality.

## Discussion

In this study we estimated the prevalence and prognostic impact of MetS in patients with COPD managed in primary care in the UK. Using a definition of MetS derived from indices routinely recorded in primary care [7], we estimated a prevalence of MetS of ∼10%. The presence of MetS was associated with 19% higher mortality over 5 years in people with COPD, but when this survival analysis was stratified by other covariates at baseline, this impact on prognosis was substantially attenuated and no longer statistically significant. Importantly, the individual components of MetS exerted differential effects on survival with obesity conferring a survival advantage, whereas the presence of hypertension or diabetes was associated with higher mortality.

Our prevalence estimates for MetS are lower than previous studies, which report rates of between 20 and 50% in people with COPD, and was reported as being 34% in a systematic review summarising such studies [2, 13]. Such differences are unsurprising given the heterogeneity of the populations studied in previous studies and variations in the definition of MetS used to establish the presence of the syndrome. Other studies on COPD have recorded the prevalence of MetS in hospital outpatient populations or in those attending pulmonary rehabilitation [14–16]. These are likely to represent populations with more severe disease or symptom burden warranting referral to hospital or rehabilitation services, and therefore a greater likelihood of metabolic comorbidity is to be expected. Studies reporting the prevalence of MetS in COPD patients of a range of severities managed in primary care are lacking, and our study therefore provides novel insight into the prevalence in this population.

We used surrogates for internationally accepted definitions for the components of MetS, for example a BMI >30 kg·m^−2^ as a marker of truncal obesity and the presence only of a coded diagnosis of hypertension or diabetes, whereas international definitions of the syndrome incorporate prospective records of blood pressure, fasting glucose, lipid profiles and waist:hip ratios [1, 3]. Some of these indices are not routinely recorded in primary care records and therefore such surrogates are a necessity if prevalence rates in primary care populations are to be estimated using electronic healthcare records. The definition used in the current study has been reported in other studies investigating MetS prevalence from CPRD, reporting rates of 26.5% in patients with benign prostatic hypertrophy [17] but only 4% in those with Barratts oesophagus or oesophageal cancer [7]. These findings emphasise the importance of the source population in determining prevalence estimates. In addition, missing data, especially for dyslipidaemia, may have impacted our prevalence estimates. Because of the lack of routine measurement of the indices comprising MetS, prevalence rates in the broader population in the UK and internationally have been uncertain but are estimated at ∼25%. In the US estimates from the National Health and Nutrition Examination survey (NHANES) put the prevalence at around a third between 2003 and 2012, over time [18].

Whilst the presence of MetS was adversely associated with mortality in the unadjusted analysis, this association disappeared when adjusted for other disease severity variables and comorbid conditions. In our sensitivity analysis, stratification for comorbidities but not disease severity, demographics or the index of socioeconomic deprivation was critical in mediating mortality. We have previously shown that indices of COPD disease severity had a greater impact than multimorbidity on 12-month survival [11], whereas in the current study investigating mortality over 5 years, the effect of MetS on survival was significantly influenced by multimorbidity but less so by disease severity (see table 5). We observed a differential effect of the individual components of MetS (in our definition) on mortality with T2DM and hypertension adversely impacting prognosis but obesity being associated with lower mortality. These observations are in line with previous literature for these conditions in COPD, in particular the protective effect of obesity on survival, termed the “obesity paradox” [6]. The epidemiological and pathophysiological mechanisms underlying the observed protective effect of obesity in COPD are incompletely understood but could relate to over-estimation of the severity of COPD in higher weight people because of the impact of obesity on symptom burden (especially breathlessness) and lung function testing [13, 19]. We observed lower rates of active smoking in people with MetS in our analysis, in line with the known association of smoking with lower body weight. As expected, active smoking was associated with higher mortality and could represent a confounding variable mediating the relationship between BMI and mortality. There may also be a more complex interplay between body compartment composition (for example regional fat distribution and muscle mass) that influences health risk and is not sufficiently captured by the simple measurement of BMI [6, 20, 21]. A major limitation of BMI is its failure to differentiate between an elevated body fat percentage and lean mass, which may influence outcomes.

A strength of the current study is the investigation of a large, unselected population of people with COPD, but we acknowledge a number of limitations from our data. Primary care data are provided from routine patient contacts conducted for the purpose of delivering healthcare rather than acquiring health data. Miscoding and missing data are therefore frequent and require bearing in mind when interpreting the data, particularly with respect to individual (rather than population) health. Examples may be miscoding of diagnoses of asthma and COPD or miscoding of “never” and “ever” smoking status. However, CPRD has been used extensively to study COPD, and the identification of our study population was undertaken using validated medcodes. Likewise, as outlined above, drawing comparisons to other population studies of MetS will be limited by the definition of the syndrome used, and we necessarily used indices that would be available in the primary care record. We note that this definition has been used in CPRD studies previously and does allow the impact of the syndrome (as defined by these indices) on health outcomes.

In conclusion, we have shown that in patients coded as having COPD in primary care, the presence of MetS is associated with lower 5-year survival but that the impact on mortality was no longer significant when the impact of COPD disease severity and other comorbidities were accounted for. The individual components of our modified definition of MetS exerted differential effects on mortality suggesting there may be limited advantage in recording the presence of MetS as an entity as the individual component conditions of the syndrome exert different effects on future health risk, and we suggest that identifying and treating these components should be the focus of clinical care rather than seeking to diagnose MetS *per se*. Understanding the impact of managing these comorbid conditions in COPD (for example through weight/diet management programmes for patients with obesity and/or diabetes) will be an important area for future research.

## Supplementary material

10.1183/23120541.00211-2022.Supp1 **Please note:** supplementary material is not edited by the Editorial Office, and is uploaded as it has been supplied by the author.
Supplementary material 00211-2022.SUPPLEMENT
