# Supplementary material for: Mortality associated with metabolic syndrome in people with COPD managed in primary care
Source: ERJ Open Res. 2022 Oct 24;8(4):00211-2022. doi: 10.1183/23120541.00211-2022 (PMC9589337; doi:10.1183/23120541.00211-2022)
Supplement: Supplementary file 1 [file 00211-2022.SUPPLEMENT.pdf]

Supplementary Appendix S1: Codelists

The following tables list the validated COPD medcodes which were used for data extraction, ICD-10 codes for diseases of the respiratory system, a sample from our list of COPD medications, codes used to detect vaccinations and example comorbidity-related codes.

Note that a medical code will have been entered by a GP or hospital consultant for a patient's medical condition but there often exists more than one possible code corresponding to the same illness description.

Table S1a. List of validated COPD medcodes and Read codes used for data extraction.

medcode	readcode	description
18476	66YL.11	COPD follow-up
10980	H322.00	Centrilobular emphysema
26306	H320.00	Chronic bullous emphysema
23492	H320z00	Chronic bullous emphysema NOS
998	H3...11	Chronic obstructive airways disease
5710	H3z..00	Chronic obstructive airways disease NOS
1001	H3...00	Chronic obstructive pulmonary disease
37247	H3z..11	Chronic obstructive pulmonary disease NOS
11287	66YM.00	Chronic obstructive pulmonary disease annual review
105457	8CMW500	Chronic obstructive pulmonary disease care pathway
45777	8CR1.00	Chronic obstructive pulmonary disease clinical management plan
45770	66Yg.00	Chronic obstructive pulmonary disease disturbs sleep
45771	66Yh.00	Chronic obstructive pulmonary disease does not disturb sleep
18621	66YL.00	Chronic obstructive pulmonary disease follow-up
38074	9Oi4.00	Chronic obstructive pulmonary disease monitor phone invite
9520	66YB.00	Chronic obstructive pulmonary disease monitoring
28755	9Oi0.00	Chronic obstructive pulmonary disease monitoring 1st letter
34202	9Oi1.00	Chronic obstructive pulmonary disease monitoring 2nd letter
34215	9Oi2.00	Chronic obstructive pulmonary disease monitoring 3rd letter
18792	9Oi..00	Chronic obstructive pulmonary disease monitoring admin
45998	66YT.00	Chronic obstructive pulmonary disease monitoring by doctor
26018	66YS.00	Chronic obstructive pulmonary disease monitoring by nurse
37371	66YD.00	Chronic obstructive pulmonary disease monitoring due
42258	9Oi3.00	Chronic obstructive pulmonary disease monitoring verb invite
794	H32..00	Emphysema
33450	H32z.00	Emphysema NOS

14798	H312100	Emphysematous bronchitis
104608	H3A..00	End stage chronic obstructive airways disease
60188	H320200	Giant bullous emphysema
42313	679V.00	Health education - chronic obstructive pulmonary disease
10863	H36..00	Mild chronic obstructive pulmonary disease
10802	H37..00	Moderate chronic obstructive pulmonary disease
104710	9NgP.11	On COPD (chronic obstructive pulmonary disease) supportive care pathway
104985	9NgP.00	On chronic obstructive pulmonary disease supportive care pathway
16410	H32yz00	Other emphysema NOS
12166	H3y..00	Other specified chronic obstructive airways disease
67040	H3y..11	Other specified chronic obstructive pulmonary disease
46578	H321.00	Panlobular emphysema
106637	9Nk7000	Seen in chronic obstructive pulmonary disease clinic
9876	H38..00	Severe chronic obstructive pulmonary disease
93568	H39..00	Very severe chronic obstructive pulmonary disease
65733	Hyu3100	[X]Other specified chronic obstructive pulmonary disease

Table S1b. ICD-10 codes used for diseases of the respiratory system.

code range	description
J00-J06	Acute upper respiratory infections
J09-J18	Influenza and pneumonia
J20-J22	Other acute lower respiratory infections
J30-J39	Other diseases of upper respiratory tract
J40-J47	Chronic lower respiratory diseases
J60-J70	Lung diseases due to external agents
J80-J84	Other respiratory diseases principally affecting the interstitium
J85-J86	Suppurative and necrotic conditions of the lower respiratory tract
J90-J94	Other diseases of the pleura
J95	Intraoperative and post-procedural complications and disorders of respiratory system, not elsewhere classified

Table S1c. COPD medications (extract from full list of 1024).

prod#	BNF chapter	drug substance	groups
8	unknown	salbutamol	SABA
17	selective beta 2 agonists	salbutamol sulfate	SABA
31	bronchodilators	salbutamol	SABA
38	corticosteroids	beclometasone dipropionate	ICS

44	corticosteroids	prednisolone	OCS
95	corticosteroids	prednisolone	OCS
99	corticosteroids	beclometasone dipropionate	ICS
235	unknown	terbutaline sulfate	SABA
282	selective beta 2 agonists	salbutamol sulfate	SABA
454	corticosteroids	budesonide	ICS
465	unknown	salmeterol xinafoate	LABA
510	selective beta 2 agonists	salbutamol sulfate	SABA
534	antimuscarinic bronchodilators	ipratropium bromide	SAMA
549	unknown	salmeterol xinafoate	LABA
555	unknown	aminophylline	THEOPH
556	unknown	salbutamol sulfate/ipratropium bromide	SABA_SAMA
557	corticosteroids	prednisolone	OCS
578	corticosteroids	prednisolone	OCS
590	theophylline	aminophylline hydrate	THEOPH
638	selective beta 2 agonists/corticosteroids	salmeterol xinafoate/fluticasone propionate	LABA_ICS
665	selective beta 2 agonists/corticosteroids	fluticasone propionate/salmeterol xinafoate	LABA_ICS
674	selective beta 2 agonists	salbutamol sulfate	SABA
696	selective beta 2 agonists	salbutamol sulfate	SABA
719	selective beta 2 agonists	salmeterol xinafoate	LABA
743	macrolides	azithromycin	AZITHRO
746	antimuscarinic bronchodilators	tiotropium bromide	LAMA
752	mucolytics	carbocisteine	MUCOLYTICS
856	selective beta 2 agonists	salbutamol sulfate	SABA

Abbreviations: AZITHRO, Azithromycin (antibiotic); BNF, British National Formulary; ICS, Inhaled corticosteroid; OCS, Oral corticosteroid; SABA/LABA, Short/long acting beta agonist; SAMA/LAMA, Short/long acting muscarinic antagonist; THEOPH, Theophylline (a muscle relaxant).

Table S1d. Medcodes used to detect vaccinations.

medcode	readcode	description
6	65E..00	Influenza vaccination
11363	6572	Pneumococcal vaccination
12104	9OX..11	Flu vaccination
12336	ZV04800	Influenza vaccination

30411	9Oo..00	Pneumococcal vaccination
36826	6572000	Pneumococcal vaccination
53198	657K..00	Booster pneumococcal vaccination
61504	657M..00	Second pneumococcal conjugated vaccination
71121	657L.00	First pneumococcal conjugated vaccination
95092	65E1.00	Second pandemic influenza vaccination
97941	65E2.00	Influenza vaccination given by other healthcare provider
104688	65ED.00	Seasonal influenza vaccination
105195	65ED000	Seasonal influenza vaccination given by pharmacist
107156	65EE.00	Intranasal influenza vaccination

Table S1e. Anaemia example codes.

medcode	readcode	description
539	D00..12	Microcytic - hypochromic anaemia
739	D21z.00	Anaemia unspecified
795	D00..00	Iron deficiency anaemias
797	D21z.13	Macrocytic anaemia of unspecified cause
882	D00..11	Hypochromic - microcytic anaemia
1668	L182500	Iron deficiency anaemia of pregnancy
1702	D21z.12	Normocytic anaemia due to unspecified cause
1771	L182.00	Anaemia during pregnancy, childbirth and the puerperium
2054	L182400	Anaemia in the puerperium - baby previously delivered
2452	D014.00	Protein-deficiency anaemia
2464	D010.00	Pernicious anaemia
2482	D011100	Vit B12 defic anaemia due to malabsorption with proteinuria
2743	D211.00	Acute posthaemorrhagic anaemia

Table S1f. Anxiety example codes.

medcode	readcode	description
636	E200.00	Anxiety states
655	E200300	Anxiety with depression
962	Eu41111	[X]Anxiety neurosis
1758	E200400	Chronic anxiety
3407	1466	H/O: anxiety state
4534	E200z00	Anxiety state NOS
4634	E200500	Recurrent anxiety
4659	E200200	Generalised anxiety disorder
5385	Eu41.00	[X]Other anxiety disorders
6221	E292000	Separation anxiety disorder
7749	Eu41211	[X]Mild anxiety depression

7999	Z4L1.00	Anxiety counselling
8205	Eu41000	[X]Panic disorder [episodic paroxysmal anxiety]

Table S1g. Cancer example codes.

medcode	readcode	description
318	B210.00	Malignant neoplasm of glottis
319	B21..00	Malignant neoplasm of larynx
779	B49..00	Malignant neoplasm of urinary bladder
780	B46..00	Malignant neoplasm of prostate
782	B....00	Neoplasms
1044	BA06.00	Neoplasm of unspecified nature of brain
1056	B5z..00	Malignant neoplasm of other and unspecified site NOS
1062	B10..00	Malignant neoplasm of oesophagus
1220	B13..00	Malignant neoplasm of colon
1599	B4A0.00	Malignant neoplasm of kidney parenchyma
1800	B141.00	Malignant neoplasm of rectum
1918	B912.00	Neoplasm of uncertain behaviour of ovary
1952	B580.00	Secondary malignant neoplasm of kidney

Table S1h. Falls-related to example codes.

medcode	readcode	description
44119	8BIG.00	Falls caused by medication
109088	9NIf.00	Seen by community falls team
55743	67ID.00	Falls advice - hip protectors advised
105823	9Og5.00	Primary health care team falls assessment defaulted
58988	8HTl.00	Referral to elderly falls prevention clinic
9951	14OC.00	At risk of falls
105686	14OC000	At high risk of falls
42670	8Hk1.00	Referral to falls service
7970	U10..00	[X]Falls
8730	TCy..00	Other falls
6815	TC...00	Accidental falls
6835	TCz..00	Accidental falls NOS
6008	16D..00	Falls

Table S1i. Kidney disease example codes.

medcode	readcode	description
1599	B4A0.00	Malignant neoplasm of kidney parenchyma
1805	K10..00	Infections of kidney
1839	7B0..00	Kidney operations

1952	B580.00	Secondary malignant neoplasm of kidney
2304	K070.00	Atrophy of kidney
2754	585B.00	U-S kidneys
2810	PD38.00	Horseshoe kidney
2991	K13z.00	Kidney and ureter disease NOS
2997	7B00.00	Transplantation of kidney
3029	L166500	Infections of kidney in pregnancy
3303	A160.00	Tuberculosis of kidney
3314	PD02.00	Congenital absence of kidney
3337	K132.00	Acquired cyst of kidney

Table S2. Social services involvement example codes.

medcode	readcode	description
4062	3875	Social services case conference
9540	9NDA.00	Report received from social services
9838	ZL79200	Referral to social services department care manager
9909	ZL79100	Referral to social services department social worker
11239	8HHB.00	Referral to Social Services
21092	ZV63211	[V]Delayed discharge - social services
28656	9b0k.00	Social services report
30534	ZLB8.00	Seen by social services department care manager
30859	ZL46300	Under care of social services occupational therapist
32592	ZLB9.00	Seen by social services department duty staff
34884	ZK4..00	Social services client assessment
40826	ZLC3300	Seen by social services department occupational therapist
43936	ZK2..00	Screening by social services department

Table S3. Candidate predictive variables and outcomes used for standard regression models.

Name	Group	Description
patid	Reference	Patient ID; the final 3 digits refer to the Practice
sex	Demographic	Gender
age	Demographic	Age rounded to the nearest year at model start date
imd	Demographic	Index of Multiple Deprivation quintile (1=least deprived, 5=most deprived)
smoking	COPD-specific	Yes, No, Ex-smoker or Missing
mrc	COPD-specific	Medical Research Council breathlessness scale 1-5
fev1pp	COPD-specific	FEV1 percent predicted

bmi	COPD-specific	Body Mass Index (kg/m^2)
n_exacs	COPD-specific	Number of exacerbations in previous year
obese	Definition	Yes/No; Essential obesity defined as body mass index (BMI) at least 30kg/m^2
hyp	Definition	Yes/No; Essential hypertension
lowhdl	Definition	Yes/No; Essential high-density lipoprotein defined as <40mg/dL (men); <50mg/dL (women)
hightg	Definition	Yes/No; Essential triglycerides defined as >150mg/dL
diabetesT2	Definition	Yes/No; Essential type 2 diabetes mellitus
liver	Comorbidity	Yes/No; Severe liver disease
kidney	Comorbidity	Yes/No; Kidney disease
psych	Comorbidity	Yes/No; Any of the following three
anx	Comorbidity	Yes/No; Treatment for anxiety
dep	Comorbidity	Yes/No; Treatment for depression
demen	Comorbidity	Yes/No; Diagnosis of dementia
frail	Comorbidity	Yes/No; Any: malnutrition, poor vision, incontinence, falls, Social Services
asthma	Comorbidity	Yes/No; Asthma
CVD	Comorbidity	Yes/No; Any of the following four
hf	Comorbidity	Yes/No; Heart failure
stroke	Comorbidity	Yes/No; Stroke
mi	Comorbidity	Yes/No; Myocardial infarction
af	Comorbidity	Yes/No; Atrial fibrillation
mskel	Comorbidity	Yes/No; Musculoskeletal, any of the following three
ra	Comorbidity	Yes/No; Rheumatoid Arthritis
oa	Comorbidity	Yes/No; Osteoarthritis
osteop	Comorbidity	Yes/No; Osteoporosis
GERD	Comorbidity	Yes/No; Gastro-oesophageal reflux disease
mets	Outcome	Yes/No; Presence of metabolic syndrome according to presenting with 3 or more components of the definition
mort_all	Outcome	All-cause mortality within three-years (30%)

Supplementary Appendix S2: Regression models

Table S4a. Univariate HR model between adjustment variables and three-year all-cause mortality.

Variable	HR	p	95% CI
genderMale	1.24	**<0.001**	1.19 – 1.29
age	1.07	**<0.001**	1.07 - 1.07
smokingEx	1.17	**<0.001**	1.09 – 1.25
smokingCurrent	1.04	0.294	0.97 – 1.12
smokingMissing	1.25	0.003	1.08 – 1.45
imd2	1.10	0.007	1.03 – 1.18
imd3	1.05	0.157	0.98 – 1.13
imd4	1.01	0.847	0.94 – 1.08
imd5	1.07	0.050	1.00 – 1.14
fev1pp	0.98	**<0.001**	0.98 – 0.98
n_exacs	1.12	**<0.001**	1.10 – 1.14
mrc2	1.43	**<0.001**	1.33 – 1.53
mrc3	2.13	**<0.001**	1.99 – 2.29
mrc4	3.11	**<0.001**	2.89 – 3.35
mrc5	5.55	**<0.001**	4.99 – 6.18
liver	1.53	**<0.001**	1.36 – 1.72
kidney	1.89	**<0.001**	1.80 – 1.99
psych	1.30	**<0.001**	1.22 – 1.39
frail	1.64	**<0.001**	1.49 – 1.80
asthma	0.90	**<0.001**	0.87 – 0.94
cvd	2.19	**<0.001**	2.10 – 2.29
mskel	1.27	**<0.001**	1.21 – 1.33
gerd	0.97	0.365	0.90 – 1.04

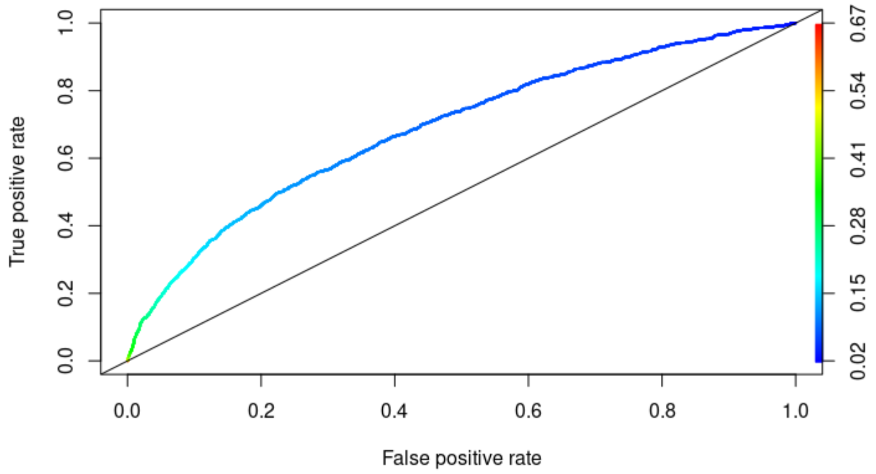

Figure S2a. Receiver Operating Characteristic on test data for logistic regression for MetS using demographic, COPD-specific and comorbidity predictors with AUC = 0.688.
